# Tumor Innervation: History, Methodologies, and Significance

**DOI:** 10.3390/cancers14081979

**Published:** 2022-04-14

**Authors:** James H. Baraldi, German V. Martyn, Galina V. Shurin, Michael R. Shurin

**Affiliations:** 1 Department of Neuroscience, University of Pittsburgh, Pittsburgh, PA 15260, USA; james.baraldi@pitt.edu; 2 Biomedical Studies Program, Chatham University, Pittsburgh, PA 15232, USA; getyn7735@gmail.com; 3 Department of Pathology, University of Pittsburgh, Pittsburgh, PA 15213, USA; shuringv@upmc.edu; 4 Department of Pathology and Immunology, Division of Clinical Immunopathology, University of Pittsburgh Medical Center, Pittsburgh, PA 15213, USA

**Keywords:** cancer, neuroimmunology, neuroimmunooncology, neurogenesis, neoneurogenesis, axonogenesis, tumor microenvironment

## Abstract

**Simple Summary:**

This comprehensive review of tumor innervation summarizes the literature from the earliest publications on the topic to the most recent. It addresses the positive and negative evidence of tumor innervation and the historical developments in thought and methodology that have led to the consensus that tumors are innervated. The role of the immune response is described, as are some important biochemical and physiological mechanisms relevant to regulation of cancer development.

**Abstract:**

The role of the nervous system in cancer development and progression has been under experimental and clinical investigation since nineteenth-century observations in solid tumor anatomy and histology. For the first half of the twentieth century, methodological limitations and opaque mechanistic concepts resulted in ambiguous evidence of tumor innervation. Differential spatial distribution of viable or disintegrated nerve tissue colocalized with neoplastic tissue led investigators to conclude that solid tumors either are or are not innervated. Subsequent work in electrophysiology, immunohistochemistry, pathway enrichment analysis, neuroimmunology, and neuroimmunooncology have bolstered the conclusion that solid tumors are innervated. Regulatory mechanisms for cancer-related neurogenesis, as well as specific operational definitions of perineural invasion and axonogenesis, have helped to explain the consensus observation of nerves at the periphery of the tumor signifying a functional role of nerves, neurons, neurites, and glia in tumor development.

## 1. Introduction

In non-pathological physiology the nervous system pervades every organ system. The presence of nerves or neurites indicates the local necessity for neural activity. Intra- and extra-cellular signals drive and sustain innervation, and absence of the same signals removes the metabolic burden of maintaining such connections [1]. The historical progression of research from psychoneuroimmunology to neuroimmunooncology has solidified the collective acknowledgment that the nervous system participates in cancer etiology. Numerous researchers have concluded that tumor growth occurs in relation to the nervous system, positing the appearance of nerves in tumors as due to perineural invasion or to axonogenesis. In perineural invasion tumors grow around existing nerves and then invade them; the nerves stimulate the tumors’ growth and provide routes for cancer cell dissemination. In axonogenesis, sometimes also called neoneurogenesis, growing nerves infiltrate tumors; this phenomenon is linked to cancer progression, as well [2]. The historical divergence of interpretations of the nature of the relationship of the nervous system with cancer etiology warrants a review of the various research approaches and their results. This is the first comprehensive review of the positive and negative interpretations of nerve identification and localization in solid tumors.

## 2. Historical Aspects

Since 1897, investigators have observed numerous instances in which it appears that nerve fibers have colocalized with neoplastic tissue, including malignant tumors, in humans and in experimental animals (Table 1) [3,4,5,6,7,8,9,10,11,12,13,14,15,16,17,18,19,20]. Central nervous system (CNS) neurons and glia may develop into CNS tumors, but most solid tumors associated with the nervous system occur outside of the CNS and receive innervation from the peripheral nervous system (PNS) [21], especially the autonomic nervous system, which has been implicated in various tissues as a regulator of cancer development and progression [22,23,24,25,26,27]. Evidence of innervation by the PNS of solid tumors has accumulated as the methodologies of identifying the phenomenon have progressed from methylene blue staining to electrophysiology to immunohistochemistry to pathway enrichment analysis. However, despite advances in technology and technique, as well as a growing acceptance of the increasingly axiomatic notion that tumors are innervated, evidence of no innervation within the tumor mass remains part of the scientific record (Table 2) [4,28,29,30,31,32,33,34,35], as do qualifications of the deceptively straightforward assertion that solid tumors are innervated. Many demonstrations of tumor innervation have been imperfect. Innervation of solid tumors has been shown to be variable in its distribution within or outside the tumor mass, and this spatial distribution has been shown to vary based on the time course of its development.

## 3. Ambiguous Evidence of Tumor Innervation

The first attempt to describe the relationship of nerves with tumors appeared in *The Journal of Experimental Medicine* in January 1897 under the sole authorship of H.H. Young, M.D., from the Anatomical Laboratory of the Johns Hopkins University. In his manuscript Young referenced previous publications, such as pathology textbooks, that remain “either entirely silent regarding this topic or [that] dismiss it with an acknowledgment of our ignorance”. In the previous year, Lücke and Zahn inferred the innervation of tumors from their clinical observations of pharmaceutical attenuation of cancer pain, but they could not distinguish the emergence of nerves in tumors as having arisen from perineural invasion or from axonogenesis [36]. Young likewise conceded an inability to make this distinction, a practical impossibility given the inherent limitations of his staining technique. His paper is remarkable as being the first to address this question and to employ the then-novel morphological visualization technique of methylene blue staining to this end. He positively identified tumor innervation in half of his samples but not in the other half, concluding that nerves appear in tumors more frequently “than has previously been suspected”. It is important to note that Young pursued the methylene blue avenue of investigation after an initial attempt with the Golgi method of silver staining afforded “such uninteresting results with these tissues that it was discarded” [3].

In 1910 an investigator surnamed Meyer, referenced by Martynow in his brief 1930 review [4], used the Bielschowsky stain, a silver staining technical improvement over the Golgi stain, to conclude that the nerve fibers that he was able to visualize were not formed by axonogenesis. The following year a Goldmann confirmed Young’s observations but went further than Young in attributing his visualized tumor nerves to axonogenesis. He also proffered an explanation of Lücke’s patients’ experiences: new tumor formation puts so much pressure on existing nerves as to obliterate them with the exception of even the smallest outgrowths of the vagus nerve, implying a function of this nerve with respect to tumor development. In 1925 a group of Japanese researchers revisited the Golgi stain, finding new nerves in benign growths but only pre-formed nerves in malignancies; a French team disputed the reliability of the methodology [6].

The focus of the researchers Itchikawa, Baum, Uwatoka, and Engle on epithelial carcinogenesis arising in response to experimental perturbations gave pause that same year (1925) to Argaud [5]. Reasoning that spontaneous cancer development follows from different histogenetic processes than those induced experimentally by Itchikawa inter alia, Argaud examined the behavior of nerve fibers in human neoplasias. Excluding those fibers enclosed by perineural invasion, and thereby perhaps making a distinction without a difference, his team considered only “the young fibers dragged along by the neoplastic guts (sic) as they lengthen, and multiplying their arborizations due to the ever-increasing number of abnormally proliferating cells” (“les fibres jeunes entraînées par les boyaux néoplasiques au fur et à mesure de leur allongement, et multipliant leurs arborisations en raison du nombre toujours plus grand des cellules anormalement prolifères”). On the basis of this morphological investigation, Argaud concluded that nerves and nerve endings grow in the epithelioma, both between its neoplastic cells and into the mass itself, and that the nerve growth corresponds to the neoplastic growth of the epithelium. Argaud regarded the fact that the epithelioma consisted of a single layer of epithelial cells as proof of his conclusion.

In his 1930 essay Horst Oertel argued for a functional role of nerves in tumors based on the premise of the indivisibility of each tissue and organ system, which must consist of a common set of characteristics [37]. “Indeed”, he wrote, “body cells are kept in contact with their outer environment entirely through their nerve and vascular supply”, including, naturally, tumors. In his brief 1930 review Martynow indicated that in both a human carcinoma and an experimental murine cancer the nerve tissue distant from the cancer exhibited no changes, whereas the nerve tissue closer to the tumor demonstrated both decay of old nerve fibers as well as formation of new ones [4]. This crucial distinction would encapsulate the theme, and begin to reconcile the contradictions, of positive and negative evidence of tumor innervation.

## 4. Methodological and Conceptual Clarifications

For half a century investigations into the question of tumor innervation were limited to histological snapshots. In 1949 Shapiro and Warren performed the first physiological investigation of tumor innervation [8]. They visualized blood vessels on and just below the surface of the tumor due to the inherent limitation of their slit-lamp microscopy visualization methodology. Because they could only visualize blood vessels on the tumor surface, and because only arterioles, as opposed to capillaries and veins, were presumed to receive motor innervation, they reasoned that finding a vessel that contracts upon sympathetic stimulation would require an arteriole to be on the tumor’s surface. They attributed the preponderance of previous negative findings, all in studies of morphology, to the fact that tumor-supplying arterioles tend to lie within the tumor, i.e., nearer the origin of the blood supply, whereas capillaries and veins can be found more prevalently on the tumor’s surface. Following tumor transplantation (carcinoma in the rabbit eye and mesothelioma in the mouse eye) and growth induced by electrical stimulation, they observed a variable degree of innervation of newly formed arterioles, which took from 3.5 to 7.5 months to develop clearly observable contractility. They attributed the likelihood of negative findings to this long time course. The investigators’ morphological observation of tumors growing into the space of the interior ocular chamber suggested that perineural invasion could not explain the presence of the nerve fibers in the tumors, which must therefore have grown with the developing tumors. This study established an unambiguous physiological observation of new nerve fibers growing within a tumor and keeping pace with it for a specific function.

Following the emergence of formal neuroendocrinology in the 1940’s [38], and with the cognitive revolution of the 1950’s and 1960’s [39,40], came studies linking psychology with cancer [41,42,43,44]. In multiple papers Lawrence LeShan emphasized the finding of the loss of a significant personal relationship by individuals who later developed neoplastic disease. The psychologist attributed the development of the etiology to an overwhelming “despair” in the patient, emphasizing the writings of the philosophers Søren Kierkegaard and Martin Buber and the poet Elizabeth Barrett Browning and speculating within a psychoanalytic framework. This psychosomatic strain of thought followed from the German and Swiss anthroposophic movement of the 1920’s to 1960’s that posited a holistic relationship between the emotions and pathophysiology, including cancer [45]. In the 1970’s Robert Ader, Nicholas Cohen, and David Felten broke ground with their work in behaviorally conditioned immunosuppression [46] to found the field of psychoneuroimmunology. Their eponymous 1981 tome posited the neuroimmune axis as a unified system of defense against insults to homeostasis, from psychological stress to viral pathogens, and it alluded to clinical and experimental reports of the onset of cancer within the context of a psychologically mediated immune response [47].

Amanda Ramirez, a psychosocial oncologist, and colleagues noted in the 1980’s that although a large body of anecdotal evidence amassed since the eighteenth century suggested a link between life events and the onset of cancer, conceptual and methodological weaknesses plagued investigation into this association [48]. Ramirez et al. (1989) [49] attempted to address these concerns by accurately identifying the date of the onset of tumor growth. They explored the association between stressful life events and relapse in operable breast cancer, finding that severely threatening life events were associated with the first recurrence, as was the relative risk of relapse. They concluded that their observational results suggested a prognostic association between severe life stressors and breast cancer, a subject that became the target of investigations into its roots in terms of neuroendocrinology [50,51,52,53] and other environmental [54], experimental [55], and clinical [56] factors. The role of psychological stress in cancer development is now widely accepted [57,58,59,60,61,62,63] and reported to be predicated on the roles of the sympathetic nervous system [64,65] and the hypothalamic-pituitary-adrenal axis in alteration of lymphocyte apoptosis, expression of cancer-survival genes, and immune response mediation [66].

## 5. Negative Evidence of Tumor Innervation

Some of the earliest investigations of tumor innervation could identify neither a specific organizational role for neurons in the tumor microenvironment [28] nor neurotrophic influence on tumors [29]. In 1958 Jabonero published a German article on his investigations into innervation of the dermatological glomus tumor [30]. Using the Bielschowsky silver stain, he reported finding no increase in the tumor of normal Hoyer-Grosser organs, thermosensitive neural bodies that dilate to maintain blood flow to the nail bed. On the contrary, he found a lower amount of innervation to such organs in the cancerous, as opposed to healthy, tissue. Jabonero also reviewed previous negative evidence of tumor innervation, which was consistent with his findings. It is worth quoting at length, not only for the coherence of the writing, but also for the identified references to the authors of these prior studies. Additionally, this is the first publication in English of this material (translated from German by JHB):

”According to Herzog, the nerve elements of spontaneous growths are mostly to be understood as preserved, preformed structures. There is said to be a noticeable resistance of the nerve elements to the tumoral proliferation, even if they occasionally show signs of degeneration. Proliferative processes, as described by some authors, have nothing to do with the tumor itself, but are merely phenomena of regeneration as a result of the abnormal elements leading to the development of experimental tumors. Oertel described a nervous plexus of the same arrangement as the nerve connections supplying the normal organ. Adventitial plexuses attached to the great blood vessels and arranged spirally around the blood capillaries can be seen. Martynkov (sic) did not find any morphological changes in the nervous elements running along the blood vessels in the tissues immediately surrounding the tumor mass. Cailliau believes that the tissue of malignant tumors possesses a special nervous system which is intimately related to the stage of development of the tumor and should be regarded as nervous stroma (neurostroma). The nervous system of the vessel walls is said to be quite different from the normal nervous supply. The innervation of the tumor tissue is more plentiful than in normal similar territories. The more malignant the neoplasm, the greater the wealth of nervous elements. With Bordallo (1948) I have described the changes in the autonomic terminal formation in rectal cancer. Between the cancer cells the nervous syncytial elements have completely perished. Changes in form, structure, and color of nervous cords occur in the immediate vicinity of the tumor. These are alterations that are not exclusively dependent on tumor proliferation but also—and perhaps mainly—on inflammatory infiltration”.

Jabonero went on to describe his findings of what was left of the innervation of the arteriovenous anastomosis of the nail bed following invasion by the tumor mass: a linear distribution of irregularly scattered granules of the disintegrated nervous tissue, thin neurofibrillary tangles, and, occasionally, clear delineation of the nerve terminal. He summarized the relationship of nerve fibers with the tumor by emphasizing that neural structures were rarely observed within the tumor mass due to their corrosion by it.

Mitchell, Schumacher, Kaiserling, and then Stauber published two similar studies in 1994 detailing their use of indirect immunohistology to identify immunoreactive nerve fibers within benign, malignant, and experimental tumors: hemangiomata, carcinomata, and experimental transplanted tumors in one study and chronic mastopathies, fibroadenomata, and breast cancers in the other study [31,32]. They found a consistent presence of nerve fibers in the vicinity of tumorous blood vessels and in stroma supporting the tumor tissue but no nerve fibers within the tumors. This finding reflected a common theme of the colocalization of nerve fibers and solid tumors: namely, that when they are found growing together, the fibers tend to be at the tumors’ peripheries and not within the centers of the masses. The investigators concluded that although major blood vessels supplying the tumor are innervated, newly formed blood vessels within a tumor are not innervated, and that, therefore, angiogenesis, a necessary component of tumor development, must be regulated by a means other than neural.

A 2001 immunohistochemistry study investigating liver carcinoma innervation likewise found evidence of the absence of nerve fibers within the tumoral sinusoids and fibrous septa of human hepatocellular carcinoma as well as a low density of innervated vasculature in the carcinoma’s capsule [33]. The same study found a very low density of nerve fibers in the tumoral stroma of intrahepatic cholangiocarcinoma. Neither carcinoma type was found to have innervation of the neovascularization, but both had some innervation of pre-existing blood vessels and extensive innervation of non-tumorous regions of the tissue. The obvious conclusion was that these carcinoma types were, essentially, not innervated.

Since 2001, various studies have found positive evidence of tumor innervation in histological assessments of human pathology as well as in experimental animal models; see the next section below. Some studies, however, continued to find evidence of no tumor innervation. For instance, two unrelated 2012 studies employed the same neuronal marker protein gene product 9.5 (PGP 9.5) as Mitchell et al. (1994) [31,32] to assess innervation patterns in two distinct tissue types in order to explain the early-stage imperceptibility of cancer growth in them. Habash et al. examined human oral squamous cell carcinoma and found no PGP 9.5 immunoreactivity in tumor-adjacent tissue in eighteen of thirty cases; pre-existing nerve fibers in tumor-adjacent tissue in twelve of thirty cases; and labeled nerve fibers in two of thirty cases. The results of this study helped to explain the painlessness of early-stage oral cancer [34]. Similarly, Tomita used PGP 9.5 to localize nerve fibers in normal human colon tissue and benign and malignant colon growths. Nerve fibers were not detected in the tumor mass, but tumor-associated neurogenesis was observed in the submucosa as well as in the highly vascularized lamina propria of benign hyperplasias (i.e., polyps and adenomata). Fine nerve fibers were observed in mucosal stroma adjacent to, but not within, T(1) carcinomata. Fragments of Auerbach’s plexus were observed in T(3) colonic tumors, but increased nerve fiber density was not. The results of this study helped to explain the “silent clinical presentation” of colonic carcinomata until its spread to other organs [35].

## 6. Positive Evidence of Tumor Innervation

The year 2001 was productive for the publication of findings demonstrating positive evidence of tumor innervation. In January 2001 Zhou et al. assessed the prevalence and location of nerves in prostate biopsy specimens with and without adenocarcinoma [9]. The investigators proceeded from the premise that absence of evidence is not evidence of absence. Namely, if nerves are consistently absent or scarce in one of three examined locations (i.e., apex, mid-gland, and base), then no evidence of perineural invasion at a given location does not necessarily equate to absence of perineural invasion throughout the cancerous area. Perineural invasion was defined as a partial or complete tight encirclement of a nerve bundle by prostatic adenocarcinoma cells. The hematoxylin and eosin (H&E) stain, the most commonly used in pathological diagnosis of biopsy specimens, was compared with the S-100 stain, used in the aforementioned study on liver carcinoma innervation, in terms of their abilities to detect nerves in cancerous and benign prostate specimens. S-100 staining found, first, even neural distribution throughout the three prostate regions with no significant difference between the numbers of nerves in them and, second, significantly more nerves than H&E staining did. Apparently consistent with studies concluding that tumors are not innervated, the nerve density in cancerous specimens was significantly smaller than that in benign specimens. However, the nerve distribution of cancerous and benign specimens was similar, a finding that the investigators interpreted to mean that a discovery of no perineural invasion may equate to a true absence of perineural invasion. This similarity of nerve distribution between cancerous and benign tissue, therefore, implies the innervation of prostatic adenocarcinoma.

Despite the history of experimental evidence of tumor innervation, however ambiguous, and a clinically relevant link between psychology and neoplastic disease, Seifert and Spitznas began the abstract of their March 2001 paper with the statement that although “[i]t is generally assumed that tumours are not innervated”, they had accidentally observed a nerve fiber within an adenoma in the eye of a patient in their native Germany [10]. These investigators used staining of tumor specimens with uranyl acetate and lead citrate and electron microscopy to identify innervation of the peripheries of two tumor types. Adenomata of both the pigmented and non-pigmented ciliary epithelia had nerve fibers embedded in Schwann cells at the tumors’ margins. No nerve fibers were identified within the tumors themselves, not even in association with the blood vessels supplying the tumors. The authors pointed out that although the tumors’ neural tissue comprises a small proportion of the tumors’ total tissue, the mere presence of nerve fibers in the tumor tissue suggests a function for the nerve fibers vis-à-vis the tumor tissue. Although their findings were similar to those of investigators that concluded that tumors are not innervated, Seifert and Spitznas concluded otherwise on the basis of the presumption that the presence of nerves is pathognomonic of their function. Seifert and others followed up this study with one on carcinoma of the human urinary bladder in order to confirm that tumor innervation is not an organ-specific phenomenon. In this second study the investigators used PGP 9.5 to visualize nerve fibers in the tumor stroma as well as to demonstrate neuronal reactivity to vasoactive intestinal peptide (VIP), confirming innervation of the bladder carcinoma [14].

An interdisciplinary team at the University of Minnesota used a mouse model to examine functional interactions between tumors and peripheral nerves that may contribute to cancer pain [11]. Cain et al. (December 2001) implanted fibrosarcoma cells into the mouse femur as a valid model of human bone cancer. Electrophysiological recordings from primary afferent fibers established a significant increase in spontaneous activity in unmyelinated, but not myelinated, fibers associated with the tumors. In a companion paper Wacnik et al. (December 2001) reported elevated release from the tumor of the peptide endothelin-1 (ET-1), the most potent vasoconstrictor known.^12^ This finding was consistent with previous studies that had implicated the peptide in the transmission of nociceptive information [67,68,69,70,71,72] as well as studies that found the peptide secreted in high concentrations by metastatic prostate and breast cancer cells [73] and in the plasma of men with prostate cancer [74].

Cain et al. also employed immunohistochemistry to make morphological observations of nerve fiber density and branching following murine fibrosarcoma implantation [11]. Their finding that the density of unmyelinated epidermal nerve fibers, which contain nociceptors, increased at first and then sharply decreased, coinciding with loss of electrophysiological activity, led them to conclude that the initial increase activated and sensitized the fibers, and that the subsequent decrease implicated neuropathic involvement. This finding was consistent with clinical data that had demonstrated an association between a reduction in cutaneous innervation and a variety of sensory neuropathies, from diabetes to HIV to post-herpetic neuralgia. Oaklander (May 2001) had suggested that cutaneous denervation led to increased activation of more-proximal sensory neurons, resulting in the hyperalgesia observed as a model of human cancer pain [75].

Ayala et al. (November 2001) sought to define “perineural invasion” in terms of a novel model system of co-cultured murine dorsal root ganglia and human prostate cancer and stromal cells, a combination intended to induce neurite outgrowth as a demonstration of the “active, specific, and reciprocal interaction” between nerves and prostate cancer cells [13]. Cell-seeding density dependence was observed, as neurite density depended on prostate cancer cell number. Neurite growth diminished following initial cancer cell contact, suggesting active neurite recruitment by the cancer cells. Cancer cells then migrated along neurites in the retrograde direction, a clear manifestation of perineural invasion, an interactive process between nerves and cancer cells that results in an increased survival advantage for cancer cells in the perineural space [76]. Not observed were interactions between stromal cells and nerves. In the years ahead Ayala and others would revisit variations on this methodology.

For instance, a 2003 study of human esophageal squamous cell carcinoma and cardiac carcinoma found not only that they were innervated by peptidergic nerve fibers, but also that they could induce neurite growth into the tumor masses [15]. Antibodies of a greater number of neuropeptides than used in previous studies (ten in this case) were used for immunohistochemical staining to identify nerve fibers. The neuropeptides used were calcitonin gene-related peptide (CGRP), galanin (GAL), substance P (SP), neurotensin (NT), somatostatin (SOM), cholecystokinin (CCK), leu-enkephalin (L-ENK), dynorphine (Dyn), neuropeptide Y (NPY), and met-enkephalin (M-ENK). Substantial distribution of the neuropeptides was observed in the midst of tumor cells, in contact with them, and even encircling them. 63% and 67%, respectively, of experimental esophageal and cardiac tumor cubic-millimeter blocks induced extension of neurite processes from chick embryo dorsal root ganglia towards, next to, and around the tumors. Although the functional role of these new fibers is not clear, their proximity to the tumor cells suggests a role of the nerve terminals in the regulation of tumor growth and/or differentiation, consistent with the recognized role of neuropeptides on tumors.

In 2008 Ayala et al. drew on their successful in vitro model, as well as from clinical evidence that spinal cord injury patients rarely develop prostate cancer [77,78], to identify increased nerve density in human prostate cancer as a consequence of axonogenesis, which they defined as nerve density enlargement, and neurogenesis, which they defined as neuron soma number increase [18]. This identification occurred by way of two- and three-dimensional reconstructions of entire prostates from aggregation of S-100-stained sections. Thus, the investigators explored spatial and temporal associations between increased nerve density, preneoplastic lesions, and neoplasias. The finding that preneoplastic lesions exhibited a higher nerve density than normal prostate tissue, but not as high as proper neoplasias, suggests that axonogenesis and neogenesis may support the initiation of prostate cancer, and that these processes precede perineural invasion.

Albo et al. (2011) used an in vitro model of co-culturing murine dorsal root ganglia with human colonic carcinoma as well as in vivo PGP 9.5 immunohistology to demonstrate the occurrence of neurogenesis [19]. The investigators observed active tumor cell migration along dorsal root ganglia dendritic projections. More-advanced disease stages were associated with higher degrees of neurogenesis and reductions in disease-free survival. Multivariate analysis found that neurogenesis was second only to the presence of metastasis in predicting a poor outcome in patients with colorectal cancer. The authors of the report made an explicit connection between their results and their recommendation to use evidence of neurogenesis to inform clinical treatment of colorectal cancer.

## 7. Neuroimmunooncology

Two 2005 studies exploring murine models of cancer pain found immunohistochemical evidence of innervation of fibrosarcoma and pancreatic adenocarcinoma, respectively [16,17]. The latter study included in its methodology an assessment of nerve growth factor expression by macrophages, and hence its discipline could be characterized not merely as “neurooncology”, but more precisely as “neuroimmunooncology”. The study in question showed clear evidence of the interaction of all three systems in driving tumor innervation.

The regression of tumorous growths after an infection or fever has been reported anecdotally since dynastic Ancient Egypt [79,80], but it was not until the late nineteenth century that the immune system was made a therapeutic target in cancer treatment. Busch (1868) deliberately infected a cancer patient with a streptococcal bacterium to induce erysipelas, an infection of the upper dermis, leading to shrinkage of the malignancy [81]. Fehleisen (1882) repeated the treatment and identified *Streptococcus pyogenes* as the causative infectious agent [82]. In 1891 Coley scaled the cure to over a thousand patients, into whom he injected heat-inactivated bacteria to treat predominantly inoperable sarcomata [83]. Poor research conduct, subsequent technological advancements in radiation therapy and chemotherapy, and changes in attitude postponed widespread acceptance of the legitimacy of the concept of immunooncology for several decades [84], during which both animal models [85] and clinical trials [86] vindicated the treatment approach. Moreover, the immune response has been found not only to treat an already developed cancer, but also to act in defense against its development in the first place. For instance, patients with immunosuppression due to organ transplantation have an increased risk of cancer development, especially of non-melanoma skin cancer [87,88].

Recent findings have implicated bidirectional nervous/immune pathways in a variety of neurological and psychiatric pathologies [89,90,91,92], and many cell types are known to contribute to a cancer-supportive tumor microenvironment, including cancer-associated fibroblasts, myeloid-derived suppressor cells, T and B regulatory cells, regulatory dendritic cells, tumor-associated alternatively activated type 2 macrophages and type 2 neutrophils, and some mast cells, all of which facilitate immunosuppression and tolerogenicity [93,94]. Neurotransmitters, particularly those involved in stress reactions, have been shown to impair the functions of several leukocyte subsets. For example, ligands of β-adrenergic receptors inhibit the cytotoxic activity of natural killer cells [95,96]; norepinephrine impairs several functions of dendritic cells [97] and inhibits the migration of neutrophil granulocytes [98]; and substance P provides an anti-adhesive signal for T cells [99]. The nervous system therefore influences the immune system, and immunosuppression by the nervous system through strong adverse experiences (e.g., chronic stress, anxiety, or depression) may facilitate tumor development [100]. Ahmad et al. have highlighted the following vicious cycle: cancer stimulates pro-inflammatory cytokines; neuroinflammation ensues; neuroinflammation hyperactivates the hypothalamic-pituitary-adrenal (HPA) axis; the dysregulated HPA axis increases cortisol; depression ensues; depression leads to immunosuppression and cancer progression [101].

## 8. Regulatory Mechanisms for Cancer-Related Neurogenesis

As mentioned above, Mitchell et al. (1994) suggested that intratumoral angiogenesis must be regulated by a means other than neural because of their finding that intratumoral neovascularization was not innervated [31,32]. Following the development of therapies targeting angiogenesis as a means to the end of limiting tumor growth [102], Entschladen et al. (2006) hypothesized that tumors may initiate their own innervation by the release of neurotrophic factors [103]. They offered as evidence the prognostic value of nerve cell markers in cancer tissue and reasoned by way of analogy with angiogenesis acting independently in cancer and in the lymphatic system. Two years later, some of the same investigators proposed the “neuro-neoplastic synapse”, through which this hypothetical process of neoneurogenesis might occur [104]. It should be noted that in normal tissue blood vessels are innervated by autonomic and sensory nerves, whereas in solid tumors blood vessels may lack normal connections and interactions with the PNS neuritis. Thus, in the tumor, the process of neoneurogenesis may not be associated with neoangiogenesis as it is in non-malignant tissues. The ensuing decade of investigations and reviews that explored this concept solidified an understanding of the role of the nervous system in the tumor microenvironment [105,106,107,108,109,110,111,112,113,114,115,116,117,118,119,120,121,122,123,124,125,126,127]. The accumulated body of knowledge, including genetic and mathematical modeling approaches, obviated the necessity for dependence on the commonly employed PGP 9.5 marker, from which prior contradictory immunohistochemical conclusions were drawn; an examination of PGP 9.5, which is sensitive for immunostaining nerve tissue, also found strong expression in the vast majority of non-neural neoplasms observed, indicating its lack of specificity as a diagnostic marker [128].

Neurogenic and angiogenic factors have some functions in common. For example, nerve growth factor (NGF) and the axonal attractant netrin-1 have angiogenic effects [129,130], while vascular endothelial growth factor (VEGF) has been implicated in axonogenesis [131,132]. Therefore, it is unlikely that only one factor is responsible for axonogenesis. This similarity of axonogenesis to the complex regulation of neoangiogenesis and lymphangiogenesis hints at a common regulation for these three processes [104]. Semaphorins, meanwhile, represent one class of neurotrophic axon guidance factors [133] that inhibit angiogenesis [134,135].

It has been shown in vitro that several neurotransmitters (viz., norepinephrine, dopamine, and substance P) greatly increase the migratory activity of carcinoma cells from the colon [136], breast [137], and prostate [138]. The intrusion of neurites into tumors and the release from the neurites of neurotransmitters provide stimuli for the migration of tumor cells and thus facilitate metastasis formation. In all assessed murine carcinoma cell lines, the β_2_ adrenergic receptor mediates the promigratory effect and the increase of metastasis formation by norepinephrine acting on PC-3 cells. Use of the nonselective propranolol [136,139] or the β_2_-selective experimental inhibitor ICI-118,551 [137,138] abolishes these effects, whereas the β_1_-specific inhibitor atenolol does not. The clinically established inhibitor metoclopramide abolishes the dopamine effect by blocking the D_2_ receptor, and the experimental L-733,060 abolishes the substance P effect by blocking the neurokinin-1 (NK_1_) receptor [138]. Norepinephrine, dopamine, and substance P induce chemotaxis; this may have relevance for the localization of metastases if tumor cells invade those organs that release these neurotransmitters. Human breast carcinoma cells migrate toward higher concentrations in graded solutions of norepinephrine, dopamine, and substance P [98,137], and human colon carcinoma cells express β_2_, D_2_, and NK_1_ receptors. The nerve endings synapsing on tumors provide neurotransmitters that facilitate metastasis formation, and they may influence other tumor cell functions, such as their proliferative activity [104].

**Figure 1:**
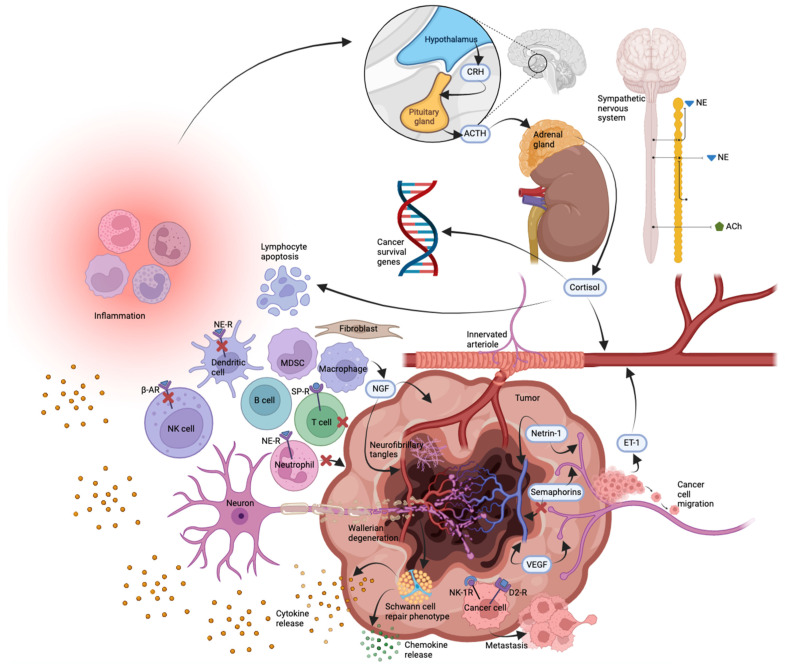
Summary of phenomena contributing to tumor innervation based on published data. Not to scale. Abbreviations: ACh (acetylcholine), ACTH (adrenocorticotropic hormone), β-AR (β-adrenergic receptor), CRH (corticotropin-releasing hormone), D2-R (dopamine receptor D_2_), ET-1 (endothelin-1), MDSC (myeloid-derived suppressor cell), NE (norepinephrine), NE-R (norepinephrine receptor), NGF (nerve growth factor), NK cell (natural killer cell) NK-1R (neurokinin-1 receptor), SP-R (substance P receptor), VEGF (vascular endothelial growth factor).

Recent evidence has implicated Schwann cells at the intersection of the neural and immune dimensions of the tumor microenvironment [94,140]. The predominant glia of the PNS, Schwann cells, associate with peripheral axons in myelinating or non-myelinating phenotypes [141,142,143,144]. Multi-layered myelination of axons by Schwann cells decreases membrane capacitance, increases electrical resistance across the axolemma, and thereby accelerates conductance of the nerve impulse [145,146]. De-differentiation of Schwann cells from the mature, myelinating phenotype to the immature, non-myelinating phenotype occurs in response to nerve injury. Peripheral nerve damage involves axon disintegration in the form of Wallerian degeneration; the once-myelinating Schwann cells respond by de-myelinating, de-differentiating, proliferating, clearing disintegrated debris, and promoting new axonal growth [147,148,149]. Schwann cells have even been observed entering the CNS to supplement oligodendrocytes’ repair of damage-induced demyelination [150]. The repair process entails Schwann cell release of chemokines, growth factors, pro-inflammatory cytokines, and factors that counterbalance pro-inflammatory cytokines. Schwann cells have been shown to direct malignant cell migration toward nerves, stimulate pancreatic and prostate cancer cell invasion, promote perineural invasion, and accelerate metastases [151,152,153,154,155]. An investigation of melanoma-induced reprogramming of Schwann cell signaling employed genetic pathway enrichment analysis to reveal the similarity between the Schwann cell repair phenotype and the Schwann cell tumor-reactive phenotype; transplanted repair-type Schwann cells were also shown to accelerate tumor growth and metastasis in vivo [156,157]. Schwann cells, then, appear to contribute to the formation of the tumor microenvironment and, therefore, to tumor development and growth. This role of Schwann cells helps to explain Martynow’s 1930 observation that tumor-adjacent nerve tissue demonstrated both decay of old nerve fibers as well as formation of new ones (Figure 1).

## 9. Conclusions

The longstanding consensus observation of nerves at the periphery of the tumor signifies a functional role of nerves, neurons, neurites, and neuroglia in tumor development. Neurotrophins and neurotransmitters act directly on receptors on the tumor tissue to promote its cellular proliferation, while the tolerogenic immune response facilitates tumorigenesis. The mechanism of neoneurogenesis consists of tumor cells releasing neurotrophins, which stimulate adjacent neurons to develop neurites that grow into the tumor. The neurons release neurotransmitters that initiate the migratory activity of tumor cells, hence tumoral invasion and cancer metastasis. Therefore, the neuroendocrine system regulates tumor cell functions and, thus, progression of cancer. Such a direct interaction explains the downregulation of the immune system by the neuroendocrine system. However, the nervous system in the presence of malignant cells may also contribute to an increase in the immune response [158]. Elucidating the relevant cellular and molecular pathways will better explain the role of neuroimmune regulation of cancer development.

Targeting the nervous, immune, and/or genetic elements of the tumor micro- and macroenvironments may constitute an effective approach to cancer therapy. Such elements include the peripheral neuroglia or tumoral genetic elements, such as alternative splice variants of tumoral mRNA. At the clinical level of analysis, the detection of nerve cell markers has been associated with poor outcomes in cancer patients, and so a better understanding of the relevance of such markers may aid in the development of therapies to improve outcomes. Clinical trials have been limited to targeting synaptic (viz., β-adrenergic) signaling. Improved understanding of the role of the sympathetic nervous system in tumor development may enhance the resolution of such targeted therapy. Better understanding of basic neurooncology may expand the therapeutic repertoire.

## Figures and Tables

**Table 1:** Observations of tumor innervation.

Cancer	Species	Method	Year	Reference
Carcinoma, epithelioma, sarcoma, myxoma, lymphoma, neuroma	Human	Methylene blue staining	1897	[3]
Cervical tumor at vagus nerve	Human	Methylene blue staining	1911	[4]
Not specified	Rabbit	Tumor implantation & sympathetic transection	1923	[4]
Various	Human, rabbit, mouse	Golgi staining	1925	[4]
Epithelioma	Rabbit	Unilateral resection of the sympathetic nervous system	1925	[4]
Epithelial meningioblastoma	Human	Apathy’s medium	1925	[5]
Squamous cell cancer	Human	Methylene blue staining	1926	[4]
Benign growth	Human	Golgi staining	1926	[4]
Not specified	Mouse, rabbit	Nerve transection	1926	[4]
Malignant neoplasm & papilloma	Mouse	Experimental coal tar	1927	[4]
Sarcoma	Rat	Sciatic nerve transplantation into transplanted sarcoma	1927	[4]
Not specified	Rat	Sympathetic nervous system resection	1927	[4]
Benign & malignant tumors	Not specified	Bielschowsky stain	1928	[6]
Malignant, cancerous, & sarcomatous tumors	Human	Silver nitrate staining	1928	[7]
Carcinoma, mesothelioma	Rabbit, mouse	Electrical stimulation	1949	[8]
Prostatic adenocarcinoma	Human	Immunohistochemistry (H&E, S-100)	2001	[9]
Adenoma	Human	Uranyl acetate & lead citrate staining, electron microscopy	2001	[10]
Fibrosarcoma	Mouse	Electrophysiological recording, immunohistochemistry (PGP 9.5)	2001	[11]
Fibrosarcoma	Mouse	Neuroendocrinology (endothelin-1)	2001	[12]
Prostatic carcinoma	Human	Dark-phase microscopy, image analysis (Optimas 6.1)	2001	[13]
Urothelial bladder carcinoma	Human	Uranyl acetate & lead citrate staining, electron microscopy, immunohistochemistry (PGP 9.5, VIP)	2002	[14]
Esophageal & cardiac carcinoma	Human	Immunohistochemistry (CGRP, GAL, SP, NT, SOM, CCK, L-ENK, Dyn, NPY, M-ENK)	2003	[15]
Fibrosarcoma	Mouse	Sleeping Beauty transposon fluorescent transfection, immunohistochemistry (PGP 9.5, CGRP)	2005	[16]
Pancreatic adenocarcinoma	Mouse	Immunohistochemistry (CGRP, neurofilament 200, tyrosine hydroxylase)	2005	[17]
Prostatic carcinoma	Human	Immunohistochemistry (S-100)	2008	[18]
Colonic carcinoma	Human	Immunohistochemistry (PGP 9.5)	2011	[19]
Prostatic carcinoma & fibrosarcoma	Mouse	Transplantation with NGF administration	2017	[20]

**Table 2:** Observations of no tumor innervation.

Cancer	Species	Method	Year	Reference
Various	Human	Bielschowsky stain	1910	[4]
Malignant growth	Human	Golgi staining	1926	[4]
Benign & malignant tumors	Not specified	Not specified	1928	[28]
Malignant neoplasms	Human	Neurotrophin assessment	1933	[29]
Glomus tumor	Human	Bielschowsky stain	1958	[30]
Hemangioma, carcinoma, experimental transplanted tumor	Mouse	Indirect immunohistology (PGP 9.5)	1994	[31]
Chronic mastopathy, fibroadenoma, breast cancer	Mouse	Indirect immunohistology (PGP 9.5)	1994	[32]
Hepatocellular carcinoma, intrahepatic cholangiocarcinoma	Human	Immunohistochemistry (S-100)	2001	[33]
Oral squamous cell carcinoma	Human	Immunohistochemistry (PGP 9.5)	2012	[34]
Colonic carcinoma	Human	Immunohistochemistry (PGP 9.5)	2012	[35]

## References

[1] Valtorta F., Leoni C. (1999). Molecular mechanisms of neurite extension. Philos. Trans. R. Soc. Lond. B Biol. Sci..

[2] Jobling P., Pundavela J., Oliveira S.M.R., Roselli S., Walker M.M., Hondermarck H. (2015). Nerve-Cancer Cell Cross-talk: A Novel Promoter of Tumor Progression. Cancer Res..

[3] Young H.H. (1897). On the Presence of Nerves in Tumors and of Other Structures in Them as Revealed by a Modification of Ehrlich’s Method of “Vital Staining” with Methylene Blue. J. Exp. Med..

[4] Martynow W. (1930). Verhalten der peripheren Nerven zum Plattenepthelkrebs des Menschen. Virchows. Arch. Pathol. Anat. Physiol. Klin. Med..

[5] Argaud M.R. (1925). Sur les terminaisons nerveuses dans les cancers humains. Comptes R. Acad. Hebd. Seances Acad. Sci. D.

[6] Itchikawa K. (1928). Sur l’existence d’une relation entre les nerfs periphériques et le développement du cancer. Bull. Assoc. Franç. Étude Canc..

[7] Oertel H. (1928). Innervation and tumour growth: A preliminary report. Can. Med. Assoc..

[8] Shapiro D.M., Warren S. (1949). Cancer Innervation. Cancer Res..

[9] Zhou M., Patel A., Rubin M.A. (2001). Prevalence and location of peripheral nerve found on prostate needle biopsy. Am. J. Clin. Pathol..

[10] Seifert P., Spitznas M. (2001). Tumours May Be Innervated. Virchows Arch..

[11] Cain D.M., Wacnik P.W., Turner M., Wendelschafer-Crabb G., Kennedy W.R., Wilcox G.L., Simone D.A. (2001). Functional Interactions between Tumor and Peripheral Nerve: Changes in Excitability and Morphology of Primary Afferent Fibers in a Murine Model of Cancer Pain. J. Neurosci..

[12] Wacnik P.W., Eikmeier L.J., Ruggles T.R., Ramnaraine M.L., Walcheck B.K., Beitz A.J., Wilcox G.L. (2001). Functional Interactions between Tumor and Peripheral Nerve: Morphology, Algogen Identification, and Behavioral Characterization of a New Murine Model of Cancer Pain. J. Neurosci..

[13] Ayala G.E., Wheeler T.M., Shine H.D., Schmelz M., Frolov A., Chakraborty S., Rowley D. (2001). In vitro dorsal root ganglia and human prostate cell line interaction: Redefining perineural invasion in prostate cancer. Prostate.

[14] Seifert P., Benedic M., Effert P. (2002). Nerve Fibers in Tumors of the Human Urinary Bladder. Virchows Arch..

[15] Lü S.-H., Zhou Y., Que H.-P., Liu S.-J. (2003). Peptidergic Innervation of Human Esophageal and Cardiac Carcinoma. World J. Gastroenterol..

[16] Wacnik P.W., Baker C.M., Herron M.J., Kren B.T., Blazar B.R., Wilcox G.L., Hordinsky M.K., Beitz A.J., Ericson M.E. (2005). Tumor-induced mechanical hyperalgesia involves CGRP receptors and altered innervation and vascularization of DsRed2 fluorescent hindpaw tumors. Pain.

[17] Lindsay T.H., Jonas B.M., Sevcik M.A., Kubota K., Halvorson K.G., Ghilardi J.R., Kuskowski M.A., Stelow E.B., Mukherjee P., Gendler S.J. (2005). Pancreatic cancer pain and its correlation with changes in tumor vasculature, macrophage infiltration, neuronal innervation, body weight and disease progression. Pain.

[18] Ayala G.E., Dai H., Powell M., Li R., Ding Y., Wheeler T.M., Shine D., Kadmon D., Thompson T., Miles B.J. (2008). Cancer-related axonogenesis and neurogenesis in prostate cancer. Clin. Cancer Res..

[19] Albo D., Akay C.L., Marshall C.L., Wilks J.A., Verstovsek G., Liu H., Agarwal N., Berger D.H., Ayala G.E. (2011). Neurogenesis in colorectal cancer is a marker of aggressive tumor behavior and poor outcomes. Cancer.

[20] Sone Y., Takatori S., Ochi E., Zamani Y., Matsuyama A., Fukuhara S., Goda M., Kitamura Y., Kawasaki H. (2017). Nerve Growth Factor Facilitates the Innervation of Perivascular Nerves in Tumor-Derived Neovasculature in the Mouse Cornea. Pharmacology..

[21] Wang W., Li L., Chen N., Niu C., Li Z., Hu J., Cui J. (2020). Nerves in the Tumor Microenvironment: Origin and Effects. Front. Cell. Dev. Biol..

[22] Magnon C., Hall S.J., Lin J., Xue X., Gerber L., Freedland S.J., Frenette P.S. (2013). Autonomic nerve development contributes to prostate cancer progression. Science.

[23] Magnon C. (2015). Role of the autonomic nervous system in tumorigenesis and metastasis. Mol. Cell. Oncol..

[24] Cole S.W., Nagaraja A.S., Lutgendorf S.K., Green P.A., Sood A.K. (2015). Sympathetic nervous system regulation of the tumour microenvironment. Nat. Rev. Cancer..

[25] Saloman J.L., Albers K.M., Rhim A.D., Davis B.M. (2016). Can Stopping Nerves, Stop Cancer?. Trends Neurosci..

[26] Kuol N., Stojanovska L., Apostolopoulos V., Nurgali K. (2018). Role of the nervous system in cancer metastasis. J. Exp. Clin. Cancer Res..

[27] Faulkner S., Jobling P., March B., Jiang C.C., Hondermarck H. (2019). Tumor Neurobiology and the War of Nerves in Cancer. Cancer Discov..

[28] Herzog E. (1928). Beitrag zur Frage der Innervation der Geschwülste. Virchows Arch. Pathol. Anat..

[29] Ryrie G.M. (1933). On the significance of nerve fibres in human malignant neoplasms. J. Pathol. Bacteriol..

[30] Jabonero V. (1958). Mikroskopische Studien über die Morphologie und die Morphopatholigie der vegetativen Innervation der menschlichen Haut (II). Acta Neurovegetativa..

[31] Mitchell B.S., Schumacher U., Kaiserling E. (1994). Are tumours innervated? Immunohistological investigations using antibodies against the neuronal marker protein gene product 9.5 (PGP 9.5) in benign, malignant and experimental tumours. Tumour Biol..

[32] Mitchell B.S., Schumacher U., Stauber V.V., Kaiserling E. (1994). Are breast tumours innervated? Immunohistological investigations using antibodies against the neuronal marker protein gene product 9.5 (PGP 9.5) in benign and malignant breast lesions. Euro. J. Cancer.

[33] Terada T., Matsunaga Y. (2001). S-100-positive nerve fibers in hepatocellular carcinoma and intrahepatic cholangiocarcinoma: An immunohistochemical study. Pathol. Int..

[34] Habash F.S., Hantash R.O.A., Yunis M.A. (2012). Assessment of the innervation pattern of oral squamous cell carcinoma using neural protein gene product (9.5)-An immunocytochemical study. J. Oral Maxillofac. Pathol..

[35] Tomita T. (2012). Localization of nerve fibers in colonic polyps, adenomas, and adenocarcinomas by immunocytochemical staining for PGP 9.5. Dig. Dis. Sci..

[36] Lücke G.A., Zahn F.W. (1896). Chirurgie der Geschwülste.

[37] Oertel H. (1930). On the mechanism of cancer development. Can. Med. Assoc..

[38] Raisman G. (1997). An urge to explain the incomprehensible: Geoffrey Harris and the discovery of the neural control of the pituitary gland. Annu. Rev. Neurosci..

[39] Miller G.A. (2003). The cognitive revolution: A historical perspective. Trends Cogn. Sci..

[40] Stanford Encyclopedia of Philosophy. Cognitive Science. https://plato.stanford.edu/entries/cognitive-science/.

[41] Kowal S.J. (1955). Emotions as a cause of cancer; 18th and 19th century contributions. Psychoanal. Rev..

[42] Reznikoff M. (1955). Psychological Factors in Breast Cancer: A Preliminary Study of Some Personality Trends in Patients with Cancer of the Breast. Psychosom. Med..

[43] LeShan L. (1961). A Basic Psychological Orientation Apparently Associated with Malignant Disease. Psychiatr. Q..

[44] LeShan L. (1966). An Emotional Life-History Pattern Associated with Neoplastic Disease. Ann. N. Y. Acad. Sci..

[45] Hitzer B., León-Sanz P. (2016). The Feeling Body and Its Diseases: How Cancer Went Psychosomatic in Twentieth-Century Germany. Osiris..

[46] Ader R., Cohen N. (1975). Behaviorally conditioned immunosuppression. Psychosom. Med..

[47] Renoux G., Biziere K., Ader R., Felten D.L., Cohen N. (1991). Neocortex Lateralization of Immune Function and of the Activities of Imuthiol, a T-cell Immunopotentiator. Psychoneuroimmunology.

[48] Ramirez A.J., Watson M., Greer S., Thomas C. (1988). Life Events and Cancer: Conceptual and Methodological Issues. Psychosocial Oncology.

[49] Ramirez A.J., Craig T.K.J., Watson J.P., Fentiman I.S., North W.R.S., Rubens R.D. (1989). Stress and relapse of breast cancer. BMJ.

[50] Thomas D.B. (1984). Do hormones cause breast cancer?. Cancer.

[51] Bosman F.T., Blankenstein M., Daxenbichler G., Falkmer S., Heitz P.U., Kracht J. (1985). What’s new in endocrine factors of tumor growth?. Pathol. Res. Pract..

[52] Vihko R., Apter D. (1989). Endogenous steroids in the pathophysiology of breast cancer. Crit. Rev. Oncol. Hematol..

[53] Olsson H. (1989). Reproductive events, occurring in adolescence at the time of development of reproductive organs and at the time of tumour initiation, have a bearing on growth characteristics and reproductive hormone regulation in normal and tumour tissue investigated decades later—A hypothesis. Med. Hypotheses.

[54] Morris S.A. (1988). Origin of mutation in neoplasia. Med. Hypotheses.

[55] Clarke R., Dickson R.B., Brünner N. (1990). The process of malignant progression in human breast cancer. Ann. Oncol..

[56] Hulka B.S. (1990). Hormone-replacement therapy and the risk of breast cancer. CA Cancer J. Clin..

[57] Levy S.M., Herberman R.B., Maluish A.M., Schlien B., Lippman M. (1985). Prognostic risk assessment in primary breast cancer by behavioral and immunological parameters. Health Psychol..

[58] Levy S., Herberman R., Lippman M., d’Angelo T. (1987). Correlation of stress factors with sustained depression of natural killer cell activity and predicted prognosis in patients with breast cancer. J. Clin. Oncol..

[59] Bovbjerg D.H. (1991). Psychoneuroimmunology. Implications for oncology?. Cancer.

[60] Cohen S., Rabin B.S. (1998). Psychologic stress, immunity, and cancer. J. Natl. Cancer Inst..

[61] Heffner K.L., Loving T.J., Robles T.F., Kiecolt-Glaser J.K. (2003). Examining psychosocial factors related to cancer incidence and progression: In search of the silver lining. Brain Behav. Immun..

[62] Costanzo E.S., Sood A.K., Lutgendorf S.K. (2011). Biobehavioral influences on cancer progression. Immunol. Allergy Clin. N. Am..

[63] Antoni M.H., Dhabhar F.S. (2019). The impact of psychosocial stress and stress management on immune responses in patients with cancer. Cancer.

[64] Wohleb E.S., Hanke M.L., Corona A.W., Powell N.D., Stiner L.M., Bailey M.T., Nelson R.J., Godbout J.P., Sheridan J.F. (2011). β-Adrenergic receptor antagonism prevents anxiety-like behavior and microglial reactivity induced by repeated social defeat. J. Neurosci..

[65] Aldea M., Craciun L., Tomuleasa C., Crivii C. (2014). The role of depression and neuroimmune axis in the prognosis of cancer patients. J. BUON.

[66] Antoni M.H., Lutgendorf S.K., Cole S.W., Dhabhar F.S., Sephton S.E., McDonald P.G., Stefanek M., Sood A.K. (2006). The influence of bio-behavioral factors on tumour biology: Pathways and mechanisms. Nat. Rev. Cancer.

[67] Raffa R.B., Jacoby H.I. (1991). Endothelin-1, -2 and -3 directly and big-endothelin-1 indirectly elicit an abdominal constriction response in mice. Life Sci..

[68] Raffa R.B., Schupsky J.J., Jacoby H.I. (1996). Endothelin-induced nociception in mice: Mediation by ETA and ETB receptors. J. Pharmacol. Exp. Ther..

[69] Davar G., Hans G., Fareed M.U., Sinnott C., Strichartz G. (1998). Behavioral signs of acute pain produced by application of endothelin-1 to rat sciatic nerve. Neuroreport.

[70] Fareed M.U., Hans G.H., Atanda A., Strichartz G.R., Davar G. (2000). Pharmacological characterization of acute pain behavior produced by the application of endothelin-1 to the rat sciatic nerve. J. Pain..

[71] Piovezan A.P., D’Orleans-Juste P., Souza G.E., Rae G.A. (2000). Endothelin-1-induced ET(A) receptor-mediated nociception, hyperalgesia and oedema in the mouse hind-paw: Modulation by simultaneous ET(B) receptor activation. Br. J. Pharmacol..

[72] Pomonis J.D., Rogers S.D., Peters C.M., Ghilardi J.R., Mantyh P.W. (2001). Expression and localization of endothelin receptors: Implications for the involvement of peripheral glia in nociception. J. Neurosci..

[73] Gokin A.P., Fareed M.U., Pan H.-L., Hans G., Strichartz G.R., Davar G. (2001). Local injection of endothelin-1 produces pain-like behavior and excitation of nociceptors in rats. J. Neurosci..

[74] Nelson J.B., Hedican S.P., George D.J., Reddi A.H., Piantadosi S., Eisenberger M.A., Simons J.W. (1995). Identification of endothelin-1 in the pathophysiology of metastatic adenocarcinoma of the prostate. Nat. Med..

[75] Oaklander A.L. (2001). The density of remaining nerve endings in human skin with and without postherpetic neuralgia after shingles. Pain.

[76] Ayala G.E., Dai H., Ittmann M., Li R., Powell M., Frolov A., Wheeler T.M., Thompson T.C., Rowley D. (2004). Growth and survival mechanisms associated with perineural invasion in prostate cancer. Cancer Res..

[77] Frisbie J.H., Binard J. (1994). Low prevalence of prostatic cancer among myelopathy patients. J. Am. Paraplegia Soc..

[78] Frisbie J.H. (2001). Cancer of the prostate in myelopathy patients: Lower risk with higher levels of paralysis. J. Spinal Cord Med..

[79] Oiseth S.J., Aziz M.S. (2017). Cancer immunotherapy: A brief review of the history, possibilities, and challenges ahead. J. Cancer Metastasis Treat..

[80] Dobosz P., Dzieciątkowski T. (2019). The Intriguing History of Cancer Immunotherapy. Front. Immunol..

[81] Busch W. (1868). Aus der Sitzung der medicinischen Section vom 13 November 1867. Berlin Klin. Wochenschr..

[82] Fehleisen F. (1882). Ueber die Züchtung der Erysipelkokken auf künstlichem Nährboden und ihre übertragbarkeit auf den Menschen. Dtsch. Med. Wochenschr..

[83] McCarthy E.F. (2006). The toxins of William B. Coley and the treatment of bone and soft-tissue sarcomas. Iowa Orthop. J..

[84] Parish C.R. (2003). Cancer immunotherapy: The past, the present and the future. Immunol. Cell Biol..

[85] Old L.J., Clarke D.A., Benacerraf B. (1959). Effect of Bacillus Calmette-Guérin infection on transplanted tumours in the mouse. Nature.

[86] Morales A., Eidinger D., Bruce A.W. (1976). Intracavitary Bacillus Calmette-guerin in the Treatment of Superficial Bladder Tumors. J. Urol..

[87] Vajdic C.M., McDonald S.P., McCredie M.R.E., van Leeuwen M.T., Stewart J.H., Law M., Chapman J.R., Webster A.C., Kaldor J.M., Grulich A.E. (2006). Cancer incidence before and after kidney transplantation. JAMA.

[88] Wimmer C.D., Rentsch M., Crispin A., Illner W.D., Arbogast H., Graeb C., Jauch K.-W., Guba M. (2007). The janus face of immunosuppression—De novo malignancy after renal transplantation: The experience of the Transplantation Center Munich. Kidney Int..

[89] Kraneveld A.D., de Theije C.G.M., van Heesch F., Borre Y., de Kivit S., Oliver B., Korte M., Garssen J. (2014). The neuro-immune axis: Prospect for novel treatments for mental disorders. Basic Clin. Pharmocol. Toxicol..

[90] Brimberg L., Mader S., Fujieda Y., Arinuma Y., Kowal C., Volpe B.T., Diamond B. (2015). Antibodies as Mediators of Brain Pathology. Trends Immunol..

[91] Heppner F.L., Ransohoff R.M., Becher B. (2015). Immune attack: The role of inflammation in Alzheimer disease. Nat. Rev. Neurosci..

[92] Hodes G.E., Kana V., Menard C., Merad M., Russo S.J. (2015). Neuroimmune mechanisms of depression. Nat. Neurosci..

[93] Li H., Fan X., Houghton J. (2007). Tumor microenvironment: The role of the tumor stroma in cancer. J. Cell. Biochem..

[94] Shurin M.R., Shurin G.V., Zlotnikov S.B., Bunimovich Y.L. (2020). The Neuroimmune Axis in the Tumor Microenvironment. J. Immunol..

[95] Lang K., Drell T.L., Niggemann B., Zänker K.S., Entschladen F. (2003). Neurotransmitters regulate the migration and cytotoxicity in natural killer cells. Immunol. Lett..

[96] Jiang X.-H., Guo S.-Y., Xu S., Yin Q.-Z., Ohshita Y., Naitoh M., Horibe Y., Hisamitsu T. (2004). Sympathetic nervous system mediates cold stress-induced suppression of natural killer cytotoxicity in rats. Neurosci. Lett..

[97] Maestroni G.J.M. (2005). Adrenergic modulation of dendritic cells function: Relevance for the immune homeostasis. Curr. Neurovasc. Res..

[98] Bastian P., Posch B., Lang K., Niggemann B., Zaenker K.S., Hatt H., Entschladen F. (2006). Phosphatidylinositol 3-kinase in the G protein-coupled receptor-induced chemokinesis and chemotaxis of MDA-MB-468 breast carcinoma cells: A comparison with leukocytes. Mol. Cancer Res..

[99] Levite M. (2000). Nerve-driven immunity. The direct effects of neurotransmitters on T-cell function. Ann. N. Y. Acad. Sci..

[100] Reiche E.M.V., Nunes S.O.V., Morimoto H.K. (2004). Stress, depression, the immune system, and cancer. Lancet Oncol..

[101] Ahmad M.H., Rizvi M.A., Fatima M., Mondal A.C. (2021). Pathophysiological implications of neuroinflammation mediated HPA axis dysregulation in the prognosis of cancer and depression. Mol. Cell. Endocrinol..

[102] Gasparini G., Longo R., Toi M., Ferrara N. (2005). Angiogenic inhibitors: A new therapeutic strategy in oncology. Nat. Clin. Pract. Oncol..

[103] Entschladen F., Palm D., Lang K., Drell IV T.L., Zaenker K.S. (2006). Neoneurogenesis: Tumors may initiate their own innervation by the release of neurotrophic factors in analogy to lymphangiogenesis and neoangiogenesis. Med. Hypotheses.

[104] Entschladen F., Palm D., Niggemann B., Zaenker K.S. (2008). The cancer’s nervous tooth: Considering the neuronal crosstalk within tumors. Semin. Cancer Biol..

[105] Strell C., Entschladen F. (2008). Extravasation of leukocytes in comparison to tumor cells. Cell. Commun. Signal..

[106] Schuller H.M. (2009). Is cancer triggered by altered signalling of nicotinic acetylcholine receptors?. Nat. Rev. Cancer..

[107] Voss M.J., Niggemann B., Zänker K.S., Entschladen F. (2010). PC-3 prostate carcinoma cells release signal substances that influence the migratory activity of cells in the tumor’s microenvironment. Cell Commun. Signal..

[108] Patani N., Jiang W.G., Mokbel K. (2011). Brain-derived neurotrophic factor expression predicts adverse pathological & clinical outcomes in human breast cancer. Cancer Cell Int..

[109] Demir I.E., Friess H., Ceyhan G.O. (2012). Nerve-cancer interactions in the stromal biology of pancreatic cancer. Front. Physiol..

[110] Li S., Sun Y., Gao D. (2013). Role of the nervous system in cancer metastasis. Oncol. Lett..

[111] Fink D.M., Connor A.L., Kelley P.M., Steele M.M., Hollingsworth M.A., Tempero R.M. (2014). Nerve growth factor regulates neurolymphatic remodeling during corneal inflammation and resolution. PLoS ONE.

[112] Colucci R., Moretti S. (2016). The role of stress and beta-adrenergic system in melanoma: Current knowledge and possible therapeutic options. J. Cancer Res. Clin. Oncol..

[113] Fernández-Nogueira P., Bragado P., Almendro V., Ametller E., Rios J., Choudhury S., Mancino M., Gascón P. (2016). Differential expression of neurogenes among breast cancer subtypes identifies high risk patients. Oncotarget.

[114] Lolas G., Bianchi A., Syrigos K.N. (2016). Tumour-induced neoneurogenesis and perineural tumour growth: A mathematical approach. Sci. Rep..

[115] Saloman J.L., Albers K.M., Li D., Hartman D.J., Crawford H.C., Muha E.A., Rhim A.D., Davis B.M. (2016). Ablation of sensory neurons in a genetic model of pancreatic ductal adenocarcinoma slows initiation and progression of cancer. Proc. Natl. Acad. Sci. USA.

[116] Zhao Y. (2016). The Oncogenic Functions of Nicotinic Acetylcholine Receptors. J. Oncol..

[117] Madeo M., Colbert P.L., Vermeer D.W., Lucido C.T., Cain J.T., Vichaya E.G., Grossberg A.J., Muirhead D., Rickel A.P., Hong Z. (2018). Cancer exosomes induce tumor innervation. Nat. Commun..

[118] Zhu Y., Zhang G.N., Shi Y., Cui L., Leng X.F., Huang J.M. (2019). Perineural invasion in cervical cancer: Pay attention to the indications of nerve-sparing radical hysterectomy. Ann. Transl. Med..

[119] Demidov V., Matveev L.A., Demidova O., Matveyev A.L., Zaitsev V.Y., Flueraru C., Vitkin I.A. (2019). Analysis of low-scattering regions in optical coherence tomography: Applications to neurography and lymphangiography. Biomed. Opt. Express..

[120] Jiang S.H., Hu L.P., Wang X., Li J., Zhang Z.G. (2020). Neurotransmitters: Emerging targets in cancer. Oncogene.

[121] Chen L., Lin J., Chen L.Z., Chen Y., Wang X.J., Guo Z.Q., Yu J.M. (2020). Perineural Invasion and Postoperative Complications are Independent Predictors of Early Recurrence and Survival Following Curative Resection of Gastric Cancer. Cancer Manag. Res..

[122] McCallum G.A., Shiralkar J., Suciu D., Covarrubias G., Yu J.S., Karathanasis E., Durand D.M. (2020). Chronic neural activity recorded within breast tumors. Sci. Rep..

[123] Hodo T.W., de Aquino M.T.P., Shimamoto A., Shanker A. (2020). Critical Neurotransmitters in the Neuroimmune Network. Front. Immunol..

[124] Mehedințeanu A.M., Sfredel V., Stovicek P.O., Schenker M., Târtea G.C., Istrătoaie O., Ciurea A.M., Vere C.C. (2021). Assessment of Epinephrine and Norepinephrine in Gastric Carcinoma. Int. J. Mol. Sci..

[125] Dlamini Z., Mathabe K., Padayachy L., Marima R., Evangelou G., Syrigos K.N., Bianchi A., Lolas G., Hull R. (2021). Many Voices in a Choir: Tumor-Induced Neurogenesis and Neuronal Driven Alternative Splicing Sound Like Suspects in Tumor Growth and Dissemination. Cancers.

[126] Liang Y., Li H., Gan Y., Tu H. (2021). Shedding Light on the Role of Neurotransmitters in the Microenvironment of Pancreatic Cancer. Front. Cell Dev. Biol..

[127] Wakiya T., Ishido K., Kimura N., Nagase H., Yoshizawa T., Morohashi S., Fujita H., Kanda T., Tatara Y., Saruwatari J. (2021). Eukaryotic initiation factor 2 signaling behind neural invasion linked with lymphatic and vascular invasion in pancreatic cancer. Sci. Rep..

[128] Campbell L.K., Thomas J.R., Lamps L.W., Smoller B.R., Folpe A.L. (2003). Protein Gene Product 9.5 (PGP 9.5) Is Not a Specific Marker of Neural and Nerve Sheath Tumors: An Immunohistochemical Study of 95 Mesenchymal Neoplasms. Mod. Pathol..

[129] Dollé J.-P., Rezvan A., Allen F.D., Lazarovici P., Lelkes P.I. (2005). Nerve growth factor-induced migration of endothelial cells. J. Pharmacol. Exp. Ther..

[130] Park K.W., Crouse D., Lee M., Karnik S.K., Sorensen L.K., Murphy K.J., Kuo C.J., Li D.Y. (2004). The axonal attractant Netrin-1 is an angiogenic factor. Proc. Natl. Acad. Sci. USA.

[131] Zhang H., Vutskits L., Pepper M.S., Kiss J.Z. (2003). VEGF is a chemoattractant for FGF-2-stimulated neural progenitors. J. Cell Biol..

[132] Han H., Yang C., Zhang Y., Han C., Zhang G. (2021). Vascular Endothelial Growth Factor Mediates the Sprouted Axonogenesis of Breast Cancer in Rat. Am. J. Pathol..

[133] de Wit J., Verhaagen J. (2003). Role of semaphorins in the adult nervous system. Prog. Neurobiol..

[134] Kessler O., Shraga-Heled N., Lange T., Gutmann-Raviv N., Sabo E., Baruch L., Machluf M., Neufeld G. (2004). Semaphorin-3F is an inhibitor of tumor angiogenesis. Cancer Res..

[135] Neufeld G., Sabag A.D., Rabinovicz N., Kessler O. (2012). Semaphorins in Angiogenesis and Tumor Progression. Cold Spring Harb. Perspect. Med..

[136] Masur K., Niggeman B., Zanker K.S., Entschladen F. (2001). Norepinephrine-induced migration of SW 480 colon carcinoma cells is inhibited by beta-blockers. Cancer Res..

[137] Drell T.L., Joseph J., Lang K., Niggemann B., Zaenker K.S., Entschladen F. (2003). Effects of neurotransmitters on the chemokinesis and chemotaxis of MDA-MB-468 human breast carcinoma cells. Breast Cancer Res. Treat..

[138] Lang K., Drell T.L., Lindecke A., Niggemann B., Kaltschmidt C., Zaenker K.S., Entschladen F. (2004). Induction of a metastatogenic tumor cell type by neurotransmitters and its pharmacological inhibition by established drugs. Int. J. Cancer..

[139] Palm D., Lang K., Niggemann B., Drell T.L., Masur K., Zaenker K.S., Entschladen F. (2006). The norepinephrine-driven metastasis development of PC-3 human prostate cancer cells in BALB/c nude mice is inhibited by beta-blockers. Int. J. Cancer..

[140] Bunimovich Y.L., Keskinov A.A., Shurin G.V., Shurin M.R. (2017). Schwann cells: A new player in the tumor microenvironment. Cancer Immunol. Immunother..

[141] Bunge R.P. (1987). Tissue culture observations relevant to the study of axon-Schwann cell interactions during peripheral nerve development and repair. J. Exp. Biol..

[142] Armati P.J., Mathey E.K. (2013). An update on Schwann cell biology--immunomodulation, neural regulation, and other surprises. J. Neurol. Sci..

[143] Whalley K. (2014). Glia: Schwann cells provide life support for axons. Nat. Rev. Neurosci..

[144] George D., Ahrens P., Lambert S. (2018). Satellite glial cells represent a population of developmentally arrested Schwann cells. Glia..

[145] Stassart R.M., Möbius W., Nave K.-A., Edgar J.M. (2018). The Axon-Myelin Unit in Development and Degenerative Disease. Front. Neurosci..

[146] Bolino A. (2021). Myelin Biology. Neurotherapeutics.

[147] Conforti L., Gilley J., Coleman M.P. (2014). Wallerian degeneration: An emerging axon death pathway linking injury and disease. Nat. Rev. Neurosci..

[148] Jessen K.R., Mirsky R. (2016). The repair Schwann cell and its function in regenerating nerves. J. Physiol..

[149] Vaquié A., Sauvain A., Duman M., Nocera G., Egger B., Meyenhofer F., Falquet L., Bartesaghi L., Chrast R., Lamy C.M. (2019). Injured Axons Instruct Schwann Cells to Build Constricting Actin Spheres to Accelerate Axonal Disintegration. Cell Rep..

[150] Harrison B.M., Gledhill R.F., McDonald W.J. (1975). Remyelination after transient compression of the spinal cord. Proc. Aust. Assoc. Neurol..

[151] Sroka I.C., Chopra H., Das L., Gard J.M.C., Nagle R.B., Cress A.E. (2016). Schwann Cells Increase Prostate and Pancreatic Tumor Cell Invasion Using Laminin Binding A6 Integrin. J. Cell. Biochem..

[152] Deborde S., Omelchenko T., Lyubchik A., Zhou Y., He S., McNamara W.F., Chernichenko N., Lee S.-Y., Barajas F., Chen C.-H. (2016). Schwann cells induce cancer cell dispersion and invasion. J. Clin. Investig..

[153] Demir I.E., Boldis A., Pfitzinger P.L., Teller S., Brunner E., Klose N., Kehl T., Maak M., Lesina M., Laschinger M. (2014). Investigation of Schwann cells at neoplastic cell sites before the onset of cancer invasion. J. Natl. Cancer Inst..

[154] Zhou Y., Shurin G.V., Zhong H., Bunimovich Y.L., Han B., Shurin M.R. (2018). Schwann Cells Augment Cell Spreading and Metastasis of Lung Cancer. Cancer Res..

[155] Martyn G.V., Shurin G.V., Keskinov A.A., Bunimovich Y.L., Shurin M.R. (2019). Schwann cells shape the neuro-immune environs and control cancer progression. Cancer Immunol. Immunother..

[156] Shurin G.V., Kruglov O., Ding F., Lin Y., Hao X., Keskinov A.A., You Z., Lokshin A.E., LaFramboise W.A., Falo L.D. (2019). Melanoma-Induced Reprogramming of Schwann Cell Signaling Aids Tumor Growth. Cancer Res..

[157] Reimand J., Isserlin R., Voisin V., Kucera M., Tannus-Lopes C., Rostamianfar A., Wadi L., Meyer M., Wong J., Xu C. (2019). Pathway enrichment analysis and visualization of omics data using g:Profiler, GSEA, Cytoscape and EnrichmentMap. Nat. Protoc..

[158] Saussez S., Laumbacher B., Chantrain G., Rodriguez A., Gu S., Wank R., Levite M. (2014). Towards neuroimmunotherapy for cancer: The neurotransmitters glutamate, dopamine and GnRH-II augment substantially the ability of T cells of few head and neck cancer patients to perform spontaneous migration, chemotactic migration and migration towards the autologous tumor, and also elevate markedly the expression of CD3zeta and CD3epsilon TCR-associated chains. J. Neural Transm..

